# KIAA0101 (OEACT-1), an expressionally down-regulated and growth-inhibitory gene in human hepatocellular carcinoma

**DOI:** 10.1186/1471-2407-6-109

**Published:** 2006-04-29

**Authors:** Minglei Guo, Jinjun Li, Dafang Wan, Jianren Gu

**Affiliations:** 1 Shanghai Medical College, Fudan University, Shanghai, China; 2 National Laboratory for Oncogenes and Related Genes, Shanghai Cancer Institute, Shanghai, China; 3 Medical School of Shanghai Jiao Tong University, Shanghai, China

## Abstract

**Background:**

Our previous cDNA array results indicated KIAA0101 as one of the differentially expressed genes in human hepatocellular carcinoma (HCC) as compared with non-cancerous liver. However, it is necessary to study its expression at protein level in HCC and its biological function for HCC cell growth.

**Method:**

Western blot and tissue array were performed to compare KIAA0101 protein expression level in paired human HCC and non-cancerous liver tissues from the same patients. Investigation of its subcellular localization was done by using dual fluorescence image examination and enriched mitochondrial protein Western blot analysis. The in vitro cell growth curve was used for examing the effect of over-expression of KIAA0101 in HCC cells. FACS was used to analyze the cell cycle pattern in KIAA0101 expression positive (+) and negative (-) cell populations isolated by the pMACSKK^II ^system after KIAA0101 cDNA transfection.

**Results:**

Western blot showed KIAA0101 protein expression was down-regulated in HCC tissues as compared with their counterpart non-cancerous liver tissues in 25 out of 30 cases. Tissue array also demonstrated the same pattern in 161 paired samples. KIAA0101 was predominantly localized in mitochondria and partially in nuclei. KIAA0101 cDNA transfection could inhibit the HCC cell growth in vitro. In cell cycle analysis, it could arrest cells at the G_1 _to S phase transition.

**Conclusion:**

KIAA0101 protein expression was down-regulated in HCC. This gene could inhibit the HCC cell growth in vitro and presumably by its blocking effect on cell cycle.

## Background

Hepatocellular carcinoma (HCC) is one of the most prevalent and lethal cancer in Asia and Africa. The development of HCC is a multi-factor in etiology, multi-step and multi-gene involvement in carcinogenesis and progression. A broad spectrum of genes have been involved in HCC development related to their genetic or epigenetic alteration, including p53[1], p16, p21[2], p27[3], beta-catenin[4], PTEN[5] and Rb etc. Recent studies on functional genomics of HCC have further revealed that a number of genes with novel sequences and unclarified functions were involved in HCC development or progression [6]. Based on cDNA array, we found KIAA0101, now designated as OEACT-1[7], as one of the genes with differential expression in HCC. In recent years, several reports described that alteration of KIAA0101 expression occurred in several cancers including thyroid [7], non-small cell lung cancer [8], and colon cancer [9]. This gene was possibly related to some mechanisms regulating cell proliferation and apoptosis [7,9].

Since the alteration of KIAA0101 expression reported so far was based on mRNA transcription, we studied the KIAA0101 protein expression level in human HCC as compared with the matched non-cancerous liver tissues by using an antibody prepared in our laboratory, and further investigated its subcellular localization in HCC cells and its biological effect on HCC growth and cell cycle.

We found that KIAA0101 was remarkably down-regulated at protein level in HCC, and it was capable of inhibiting cell growth and blocking the transition from G1 to S phase in cell cycle.

## Methods

### Tissue samples and tissue array

The human liver cancer samples and matched adjacent liver tissues were collected from the First Affiliated Hospital of Zhejiang University (Hangzhou, PR China). The HCC cell lines were provided by our lab and cultured in standard conditions (10% fetal bovine serum, 5% CO_2_). Tissue array was prepared by our lab including 161 pairs of liver cancerous tissues and adjacent non-cancerous tissues, 13 liver cirrhosis tissues and 10 normal livers. All samples of collection were under consensus agreements, and were approved by the Ethical Review Committee of the World Health Organization Collaborating Center for research in Human Production.

### Antibody preparation

KIAA0101 coding sequence was subcloned into the pEGFP plasmid and pET-32a prokaryotic expression vector (His tag) separately and sequenced for confirmation. The primers for pEGFP vector are 5-ggagatctaacatggtgcggactaaag-3 and 5-gggtcgaccattctttttcatcatttg-3; the primers for pET-32a are 5-ggggatccgcaacatggtg cggactaaag-3 and 5-ggctcgagttctttttcatcatttg-3. The sequence underlined is restriction site for subcloning. Then the pET-32a+0101 recombinant expression plasmid was transformed into competent BL21 cells. Following induction of BL21 cells with 0.4 mM IPTG at 37°C for 4 hours, then the whole cells were lysised by the suspersonic equipment. The supernatant including the cell whole proteins were flowed through the Ni-coated column and the His-KIAA0101 fusion proteins were bound. After three times wash of the column, the His-KIAA0101 fusion protein was obtained by the elution buffer and was used for rabbit polyclonal antibody production according to the standard procedure.

### Western blotting

The liver tumor and non-tumor samples were lysised in T-PER tissue protein extraction reagent (PIERCE) containing proteinase inhibitor cocktail (Roche) in 4°C for 30 minutes. The debris was discarded and the supernatant including whole proteins were quantified by BCA kit (PIERCE) and subjected to 15% SDS-PAGE (40 ug). Next the proteins were transferred onto a nitrocellulose membrane (Schleicher & Schuell BioScience) and this membrane was blocked by the 5% milk dissolved in PBST for an hour. After incubation with the fist antibody and second antibody sequencely, detection was performed by using an ECL system (PIERCE). The rabbit anti-KIAA0101 antibody was used (1:1000) and GFP antibody (1:1000). Anti-β-actin antibody (1:10000, Sigma) was used as internal control.

### Immuno-histochemistry

The tissue array plate was blocked with 3% BSA 37°C for 1 hour, then probed with KIAA0101 antibody (1:100) at 4°C in a humidified chamber for 12 hours, followed rinsed three times by PBS and incubated with secondary rabbit antibody for 1 hour at 37°C (1:1000). This antibody complex was visualized by 3, 30-diaminobenzidine (Zymed Laboratories Inc.). Sections were counterstained with hematoxylin for 5 min and mounted with Eukit (Calibrated Instrument). The result was observed under a light microscope, and a semi-quantitative score for intensity was given to the different samples: +++, ++, + and -.

### Dual immuno-fluorescence and enriched mitochondrial protein Western blot

Cultured HCC cells were seeded on the cover slips and incubated for 12 hours. Then the cells were probated with IgG-purified anti-KIAA0101 primary antibody (1:100) for half an hour in 37°C, after three times wash by PBS, the cells were incubated with TRITC-conjugated goat anti-rabbit secondary antibody for an hour. Also the same cover slip were washed and antibody angnist cytochrome oxidase I was added at 1 mg/ml in block buffer, followed by FITC-conjugated goat anti-mouse IgG at 15 mg/ml in block buffer. The cells were viewed by epi-fluorescence with Axioskop 2 universal microscope. Digital images were captured using a CCD camera and analysized by ISIS system.

For enriched mitochondrial protein Western blot, cells were rinsed twice with cold PBS and then lysed in radio-immune precipitation (RIPA) buffer for 15 min at 4°C. Cell lysates were clarified by centrifugation at 10,000 rpm for 10 min, and the supernatant was incubated with 1 ug anti-mitochondrial antibody (Neomarks) for overnight in 4°C. Then the immune complexes were precipitated by protein A/G-Sepharose beads and washed four times with RIPA buffer, followed separated by 15% SDS-PAGE electrophoresis and for the KIAA0101 Western blot.

### Co-transfection and separation of transient expression cells

Transfection was performed by incubating cells with pcDNA3.1/A+0101 together with the pMACS KK^II ^plasmid (5:1) (Miltenyi Biotec, Auburn, CA, USA). pMACS KK^II ^plasmid could be provided for the magnetic isolation of transiently transfected mammalian cells. Briefly, 4*10^6 ^cells in 6-cm plate were incubated for 5 hours at 37°C with 5 ug of the plasmid pcDNA3.1/A+0101 and 1 ug of pMACS KK^II ^magnetic plasmid in a total of 1 ml DMEM. The cells were then washed and incubated overnight in DMEM containing 10% FCS at 37°C in 5% CO_2 _atmosphere. 48 hours later, the cells were labeled by MACSselect KK^II ^microbeads and selected by MACSselect KK^II ^column according to the protocol recommended. At the same time, the co-transfection of pcDNA3.1/A with pMACS KK^II ^plasmids into cells is performed as control.

### Cell growth analysis

KIAA0101 transiently expression positively (+) cells isolated by the MACSselect KK^II ^microbeads and negative cells were seeded into 96-well flat-bottom microtiter plates equally. Cells were cultured in DMEM containing 10% FBS. Each day, 10 ul of CKK-8 solution (Dojin, Osaka, Japan) were added to each well and incubated for 2 hrs at 37°C. Optical density was read at 450 nm in a microplate reader.

### Cell cycle analysis

KIAA0101 transiently expression positively (+) cells isolated by the MACSselect KK^II ^microbeads and negative cells were fixed in PBS/70% EtOH at -20°C overnight. On the following day, cells were washed in PBS, stained with propidium iodide (50 mg/ml) and RNAse (360 mg/ml) for 30 min at 37°C, then with just propidium iodide (50 mg/ml) in PBS for at least 1 h at 4°C. DNA content was quantified using a FACSCAN (Becton Dickson). This experiment was repeated three times.

## Results

### Preparation of polyclonal antibody against KIAA0101 and test of its specificity

**Figure 1:**
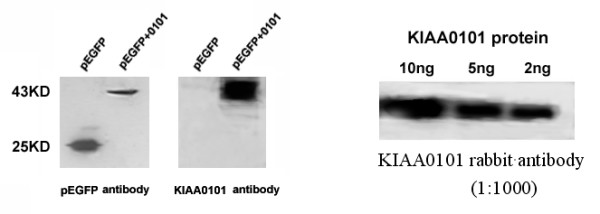
Western blot for testing the specificity of prepared rabbit KIAA0101 antibody. A: the recombinant pEGFP+0101 and pEGFP plasmids were transfected into cells separately. 48 hours later, the transfected cell protein were extracted for Western blot analysis with GFP antibody (1:1000) and prepared rabbit KIAA0101 antibody (1:1000) respectively. B: the KIAA0101 prokaryotic expression protein was loaded at different quantity (10 ng, 5 ng and 2 ng) and reacted for the test of the prepared KIAA0101 antibody specificity.

As the antibody for KIAA0101 was not commercial available, we prepared the polyclonal rabbit antibody against full-length KIAA0101-His fusion protein. The pEGFP+0101 recombinant plasmid was constructed and transfected into HCC cells. The proteins of the transfected cells were extracted for Western blot by using the anti-GFP antibody and KIAA0101 rabbit antibody to test the expression of EGFP and KIAA0101. As illustrated in Fig 1, a band of EGFP-KIAA0101 fused protein could be revealed by both the anti-GFP and anti-KIAA0101 antibodies. On the contrary, no band was observed for pEGFP vector-transfected cell protein by anti-KIAA0101 antibody, though it was positive by anti-GFP antibody. Also we used the KIAA0101 prokaryotic expression for the test of its antibody specificity. The KIAA0101 antibody could detect a specific band with the predicted size (15 KD) and the intensity of band signal was generally proportional to the amount of loaded protein. Therefore, we concluded that our prepared rabbit polyclonal antibody for KIAA0101 was specific, and it could be used for further experiments.

### KIAA0101 protein was down-regulated in HCC

**Figure 2:**
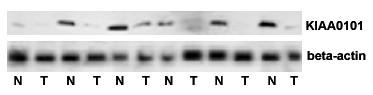
Western blot for KIAA0101 in HCC and counterpart non-cancerous liver tissues. To investigate KIAA0101 protein expression level, 30 pairs of tumor and non-tumor liver tissues were analyzed. The KIAA0101 protein expression was lower in 25 out of 30 HCC tissues as compared with non-cancerous liver tissue (N: non-cancerous tissue; T: tumor tissue). Beta-actin was used an internal control.

**Figure 3:**
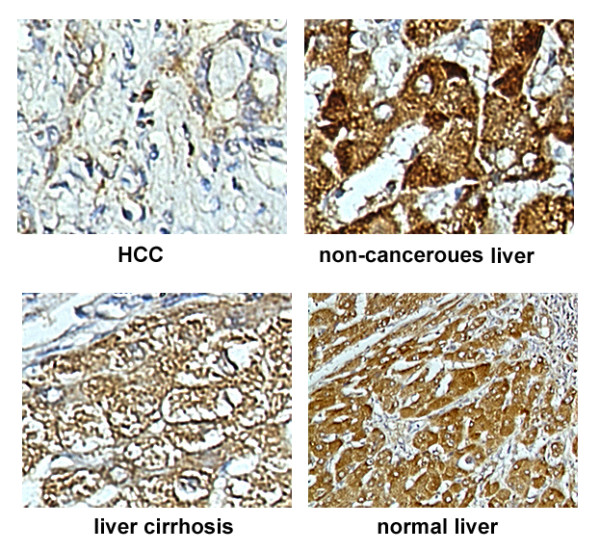
KIAA 0101 expression in tissue array was analyzed by immunohistochemistry (ISH) examination. The tissue array showed different levels of KIAA0101 protein expression in paired HCC and non-cancerous tissues, liver cirrhosis and normal liver tissues by ISH method. Weak expression of KIAA0101 was observed in HCC; while strong expression in its counterpart non-cancerous liver tissue. In 8 out of 13 liver cirrhosis and all 10 normal liver tissues, KIAA0101 protein expression level were strong(+++) or moderate(++) level.

To demonstrate KIAA0101 expression pattern at the protein level in human HCC, 30 human HCC (T) and matched noncancerous liver tissues (NT) from the same patients were analyzed by Western blot (Figure 2). The protein band intensity was compared after being normalized with that of the beta-actin internal control. It was striking that the protein expression level of KIAA0101 in HCC tissues was reduced as compared with that of non-cancerous tissues among 25 out of 30 paired samples. We have conducted the tissue array by immunohistochemical survey(ISH) for further validation. Fig 3 illustrated the different intensity between HCC and matched non-cancerous tissues, liver cirrhosis and normal liver tissues by ISH method. As demonstrated in table 1 (see Additional file 1), the tissue array showed the signal intensity was weak(+) or negative(-) in 107 out of 161 HCC tissues; while the weak (+) or negative (-) signals were seen in only 24 out of 161 samples of non-cancerous liver tissues. Therefore, the KIAA0101 protein expression was much lower in the HCC tissues as compared with the non-cancerous tissues (p < 0.01). In addition, in 8 out of 13 of liver cirrhosis tissues and all 10 normal liver tissues, KIAA0101 protein was expressed varying from moderate(++) to high(+++) level. However, the KIAA0101 protein level in HCC tissues was obviously reduced. Furthermore, we have attempted to investigate if the down-regulation of KIAA0101 protein expression had any correlation with the patients' background information, such as age, sex, histopathological grading, tumor size and HBV infection. However, we have not found any significant association between the clinical or pathological state of HCC with the KIAA0101 expression (see Additional file 2).

### Localization of KIAA0101 in mitochondria

**Figure 4:**
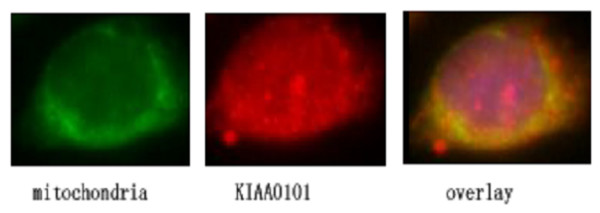
Localization of KIAA0101 predominantly in mitochondria. HCC SMMC 7721 Cells were seeded on slip for dual immunofluorescence analysis. Primary antibodies for cytochrome oxidase I and KIAA0101 were detected using FITC- and TRITC- conjugated secondary antibodies, respectively. Yellow fluorescence in the overlay image indicated the co-localization of the red fluorerscence of KIAA0101 with the green fluorescence of mitochonriion specific of cytochrome oxidase I.

**Figure 5:**
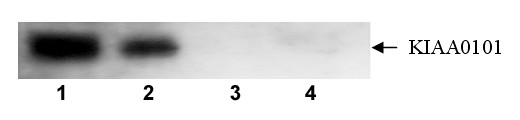
Western blot for KIAA0101 protein in mitochondrial-enriched protein fraction and total cell proteins (1 and 2 were mitochondrial enriched proteins from SMMC 7721 and HepG2 respectively; 3 and 4 were total cell protein from SMMC 7721 and HepG2 respectively).

The bioinformatics has implicated that KIAA0101 was possibly localized in mitochondrial. To verify this prediction, we have performed immuno-fluorescence survey on SMMC7721 cells by using the polyclonal rabbit antibody. KIAA0101 immuno-fluorescence was exhibited in a speckled pattern in the cytoplasm. The distribution of KIAA0101 immuno-fluorescence appeared to be similar to the distribution of mitochondria. In dual-labeling experiments, KIAA0101 immuno-fluorescence partially coincided with the image generated by a labeled monoclonal antibody that recognized cytochrome oxidase subunit I, an inner mitochondrial membrane protein. The partially co-localization of KIAA0101 with mitochondrial proteins suggested that KIAA0101 was localized predominantly in mitochondria (Fig 4). Meanwhile, we used the specific mouse anti-mitochondrial (Neomarkers) to enrich the mitochondrial proteins from total cell proteins preparation. After precipitated by the protein A/G beads, the enriched mitochondrial proteins were subjected to Western blot with KIAA0101 rabbit antibody. Fig 5 showed that there were strong signal in lanes loaded by enriched mitochondrial proteins; however, there were only very weak signal in the un-sorted total cell proteins. From these two different experiments, we could conclude that the KIAA0101 protein was predominantly located in the mitochondria of human HCC SMMC 7721 cells.

### Cell growth and cell cycle analysis

**Figure 6:**
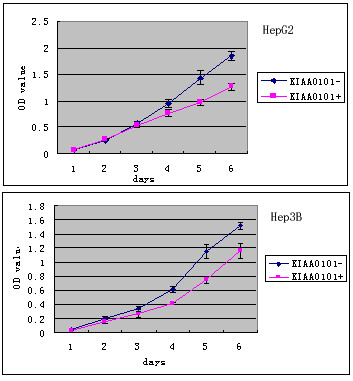
Transient expression of KIAA0101 protein inhibited the HCC cell growth in virto (p < 0.05). pcDNA3.1/A+0101 and pMACSKK^II ^plasmids were co-transfected into the HepG2 and Hep3B cells. Meanwhile, the pcDNA3.1/A and pMACSKK^II ^plasmids were co-transfected and isolated as control. After about 48 hours, the KIAA0101 expression positive cells were selected by magnetic microbeads through column and seeded into 96-well plates. At every 24 hours, the cells' growth absorbance was measured with CCK-8 kit.

**Figure 7:**
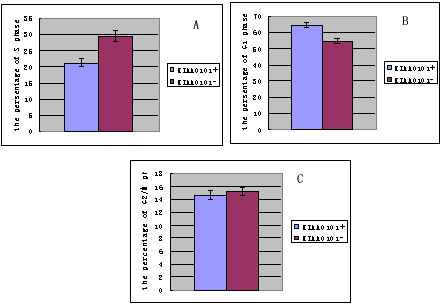
The percentage of G0/G1, S and G2/M phase in the isolated KIAA0101 expression positive (+) cells and negative cells. The KIAA0101 transiently expressed cells were separated by the magnetic beads through the pMACS column and analyzed by the FACS. At the same time, the KIAA0101 expression negative cells were done as control. A: The percentage of S phase in KIAA0101 positive (+) cells is 21.35% ± 1.28; while in negative cells the proportion of S phase is 29.45% ± 1.45 (P < 0.05). B: The percentage of G0/G1 phase in KIAA0101 positive (+) cells is 64.38% ± 1.46; while in negative cells the proportion of phase G0/G1 is 54.7% ± 1.43 (P < 0.05). The percentage of G2/M phase in KIAA0101 positive (+)cells is 14.69% ± 0.62; while in negative cells the proportion of G2/M phase is15.21% ± 0.51. These results indicated the KIAA0101 could inhibit cell cycle transition from G1 to S phase.

For better understanding if the KIAA0101 has any effect on cell growth, we employed the pMACS KK^II ^system to isolate the KIAA0101 over-expressed cells after transient KIAA0101 cDNA transfection into two different HCC cell line: Hep3B and HepG2. The isolated KIAA0101 expression positive (+) cells and the KIAA0101 negative (-) control cells were seeded into the 96-well plate. We measured the absorbance at the 450 nm with the CCK-8 kit each day. As illustrated in Fig 6, the KIAA0101 over-expression could inhibit the growth of both HCC cell line: HepG2 and Hep3B (P < 0.05). Meanwhile, as indicated in Fig 7A, the sorted KIAA0101 expression positive and negative cells were analyzed by FACS and the results demonstrated that the cell population at S phase of KIAA0101 positive cells was reduced by nearly 8% as compared with the negative cells and the difference had statistical significance(P < 0.05). The percentage of G0/G1 phase in KIAA0101 positive (+) cells is 64.38% ± 1.46, while in negative cells the proportion of phase G0/G1 is 54.7% ± 1.43. The difference was of statistical significance (p < 0.05, Fig 7B). The percentage of G2/M phase in KIAA0101 positive (+) cells is 14.69% ± 0.62 while in negative cells the proportion is 15.21% ± 0.51 with no statistical difference (Fig 7C). Taken together, the FACS result was well consistent with the retardation of HCC cell growth after over-expression of KIAA0101 protein.

## Discussion

KIAA0101 has been recently reported as a novel gene related to thyroid [7], non-small cell lung carcinoma[8] and colon cancer[9,10]. In these reports, the alteration of expression at mRNA level was documented, either up-regulated [7-9] or down-regulated [10] in cancers described. The data presented here is the first time to demonstrate the alteration of KIAA0101 expression at protein level in human HCC by using Western analysis of 30 paired human HCC and non-cancerous liver tissues. KIAA0101 down-regulated expression at protein level was observed in 25 HCC tissues (83%). Utilizing tissue array and immunohistochemical survey, it was also demonstrated that no (-) or very weak (+) signals were detected in 107 HCC tissues (T) of 161 paired HCC (T) and non-cancerous (NT) tissues (66.4%). These data demonstrated that KIAA0101 expression at the protein level was remarkably down-regulated in HCC based on both Western and tissue array analysis.

We have attempted to correlate the expression status of KIAA0101 with the clinic-pathological background and HBV infection. However, we have not obtained positive association between KIAA0101 expression and etiology-clinial state(see Additional file 2). It implicated that KIAA0101 might be presumably one of the genes essential for cell growth basic machinery which was less dependent on different etiological or differentiation status. However, the further correlation study should be necessary to survey in a much larger scale of case population.

For better understanding the possible biological effect of KIAA0101 relevant to the growth behavior of cells, we examined the effect of over-expression of KIAA0101 after transfection into two different cell lines using growth curve assays. Our data indicated an inhibiting effect on cell growth of both these HCC cell lines. These results seemed not apparently consistent with the previous reports in thyroid cancer[7] and HEK293 cells[9]. The KIAA0101 mRNA expression in thyroid cancer was up-regulated and knock down of its expression by siRNA could retard the cell growth [7], while the over-expression of KIAA0101 by cDNA transfection in HEK293 cells had no effect on cell growth on this immortalized human renal cells[9]. Our data did demonstrate that KIAA0101 over-expression could retard the cell growth of human HCC cells. These data were further supported by the results of cell cycle analysis which indicated the arrest at G_1_/S transition in KIAA0101 over-expressed cells. The inconsistency between our data and those from other laboratories might be attributed to different types of cancers or cells used for studies. It is probable that KIAA0101 might have different expression pattern in various types of cancers. Also the possibility that KIAA0101 may play various biological roles in different types of normal and cancer cells could not be excluded. Moreover, the discrepancy of data between level of mRNA and protein expression in the same tissue has been reported[11]. In chen's report, they compared the level of mRNA with protein expression among 165 proteins. A subset of proteins demonstrated a negative correlation with the mRNA expression value. Thus the possibility that a negative correlation existed between KIAA0101 mRNA with protein level could not be excluded. It may also interpret, at least in part, the controversial data about mRNA and protein expression of KIAA0101 genes from different laboratories.

In our observation, the KIAA0101 gene product was predominantly localized in mitochondria of human HCC cells, but the minor portion was found in nuclei. A recent report by Simpson F etal has described that KIAA0101 was predominantly found in mitochondria of human renal HEK293 cells, but it could translocate into nuclei after UV exposure. It was found that KIAA0101 could bind to PCNA and a putative tumor suppressor[9]. Therefore, the KIAA0101 may have a complex feature in its signal pathway and further studies of KIAA0101 are needed to explore its signal pathway in human HCC cells.

Taken together, our findings may put some insight on further elucidation of KIAA0101 in regulatory mechanism of cell growth and its relevance to HCC development and progression.

## Conclusion

This is the first report to demonstrate the down-regulation of KIAA0101 at protein level in human HCC and its over-expression could inhibit the human HCC cell growth.

## Abbreviations

HCC: hepatocellular carcinoma; FACS: flow-asisted cell sorting; ISH: in situ hybridization; MACS: magnetic activated cell sorting.

## Competing interests

The author(s) declared they have no competing interests.

## Authors' contributions

All the authors contributed to the conception during the initial stages and study design, and in the analysis and interpretation of the data, as well as to the drafting and critical revision of the important intellectual content. All the authors agreed to the final approval of the version to be published. Professor Jianren Gu was in charge of the general supervision of this research. The coauthors declare the order of authorship was based on a joint decision.

## Pre-publication history

The pre-publication history for this paper can be accessed here:



## Supplementary Material

Additional File 1 **Table 1**: The KIAA0101 protein expression in HCC, non-cancerous liver tissues, liver cirrhosis and normal liver tissues. The KIAA0101 protein expression was investigated in 161 pairs of tumor tissues and their counterpart non-cancerous liver tissue, 13 liver cirrhosis and 10 normal liver tissues by ISH. The results showed that in 107/161 tumor tissues, the intensity of KIAA0101 protein expression was + or -; while only in 24/161 non-cancerous liver tissues, the signal intensity was + or - (P < 0.05). (NT: non-tumor tissue; T: tumor tissue; LC: liver cirrhosis; NL: normal liver tissue). Click here for file

Additional File 2 **Supplement Table 1**. Comparison of KIAA0101 expression level and serological HBV markers in HCC patients Serological HBV markers included HBsAg, HBcAb, HBeAg, HbsAb, HBeAb and others. HBsAg (+), HBcAb (+), HBeAg (+) indicated patients as HBV (+). Statistical analysis indicated that the KIAA0101 (-)/(+) versus (++)/(+++) groups had no statistical difference between HBV(+) and HBV (-) patients. **Supplementary Table 2**. Expression of KIAA0101 in HCC with different histopathologicalgrades. The histopathological grading was according to standard of childpugh. The difference in intensity of expression of KIAA0101 in (-)/(+) versus (++)/(+++) groups in different histopathological grades had no statistical significance. Click here for file
