# Supplementary material for: KIAA0101 (OEACT-1), an expressionally down-regulated and growth-inhibitory gene in human hepatocellular carcinoma
Source: BMC Cancer. 2006 Apr 29;6:109. doi: 10.1186/1471-2407-6-109 (PMC1483895; doi:10.1186/1471-2407-6-109)
Supplement: Additional File 1 — Table 1: The KIAA0101 protein expression in HCC, non-cancerous liver tissues, liver cirrhosis and normal liver tissues. The KIAA0101 protein expression was investigated in 161 pairs of tumor tissues and their counterpart non-cancerous liver tissue, 13 liver cirrhosis and 10 normal liver tissues by ISH. The results showed that in 107/161 tumor tissues, the intensity of KIAA0101 protein expression was + or -; while only in 24/161 non-cancerous liver tissues, the signal intensity was + or - (P < 0.05). (NT: non-tumor tissue; T: tumor tissue; LC: liver cirrhosis; NL: normal liver tissue). [file 1471-2407-6-109-S1.doc]

|  | **Singal Intensity** | | | | **Total** |
| --- | --- | --- | --- | --- | --- |
|  | +++ | ++ | + | - |
| **NT** | 91 | 46 | 17 | 7 | 161 |
| **T** | 25 | 29 | 35 | 72 | 161 |
| **LC** | 5 | 3 | 2 | 3 | 13 |
| **NL** | 6 | 4 |  |  | 10 |
