# Supplementary material for: KIAA0101 (OEACT-1), an expressionally down-regulated and growth-inhibitory gene in human hepatocellular carcinoma
Source: BMC Cancer. 2006 Apr 29;6:109. doi: 10.1186/1471-2407-6-109 (PMC1483895; doi:10.1186/1471-2407-6-109)
Supplement: Additional File 2 — Supplement Table 1. Comparison of KIAA0101 expression level and serological HBV markers in HCC patients Serological HBV markers included HBsAg, HBcAb, HBeAg, HbsAb, HBeAb and others. HBsAg (+), HBcAb (+), HBeAg (+) indicated patients as HBV (+). Statistical analysis indicated that the KIAA0101 (-)/(+) versus (++)/(+++) groups had no statistical difference between HBV(+) and HBV (-) patients. Supplementary Table 2. Expression of KIAA0101 in HCC with different histopathologicalgrades. The histopathological grading was according to standard of childpugh. The difference in intensity of expression of KIAA0101 in (-)/(+) versus (++)/(+++) groups in different histopathological grades had no statistical significance. [file 1471-2407-6-109-S2.doc]

Supplementary table I

| singal inteinsity of KIAA0101 protein expression | HBV serological markers | | | |  | |
| --- | --- | --- | --- | --- | --- | --- |
|  | HBV (+) |  | HBV(-) |  | | Total case |
|  | case No. |  | case No. |  | |  |
| (-) , (+) | 63 (70%) |  | 27 (30) |  | | 90 |
| (++), (+++) | 27(65.6%) |  | 13 (34.4%) |  | | 41 |
| Total | 91 |  | 40 |  | | 131 |

Supplementary table II

| singal inteinsity of KIAA0101 protein expression | Histpopathologcal Grade | | | | |
| --- | --- | --- | --- | --- | --- |
|  |  |  |  |  |  |
|  | I | II | III | IV | Total (No.) |
| (-), (+) | 9 | 33 | 61 | 4 | 107 |
| (++), (+++) | 4 | 18 | 31 | 1 | 54 |
| Total | 13 | 51 | 92 | 5 | 161 |
